# 2242 Evaluation of a clinical investigation curriculum: Post-graduate outcomes

**DOI:** 10.1017/cts.2018.208

**Published:** 2018-11-21

**Authors:** Julie H. Shakib, Carol Sweeney, Jodi Cullum, Ruben Rocha, Anthea Letsou

**Affiliations:** The University of Utah School of Medicine

## Abstract

OBJECTIVES/SPECIFIC AIMS: Many CTSA programs have implemented curricula leading to clinical investigation master’s degrees. Evaluation of long-term outcomes for graduates can support curriculum improvement. METHODS/STUDY POPULATION: We evaluated graduates 1–3 years post completion of an MS in Clinical Investigation at the University of Utah. We administered the 12-item Clinical Research Appraisal Inventory (CRAI-12) describing confidence in ability to perform research tasks; we derived 6 CRAI sub-scales. Additional questionnaire items assessed current engagement in research, including percent of effort devoted to research and level of involvement in research projects using specific research methods. RESULTS/ANTICIPATED RESULTS: Graduates reported high confidence for the CRAI domain of reporting, interpreting, and presenting (on a scale of 0–20, mean 17.9±SD 1.9) and the domain of conceptualizing and collaborating (16.5±2.2) on research projects; confidence was somewhat lower in the domains of planning (14.6±3.3) and funding (14.9±2.8) projects. Graduates’ estimated current professional effort devoted to research had a median of 32%, interquartile range (IQR) 20%–70%; among graduates with clinical responsibilities, median effort devoted to research was 23%, IQR 15%–45%. In total, 74% of graduates reported moderate or high involvement in research using existing large databases, 46% reported moderate or high involvement in comparative effectiveness research, and 54% reported moderate or high involvement in quality improvement. DISCUSSION/SIGNIFICANCE OF IMPACT: A majority of clinical investigation graduates remain engaged in research but most are able to devote less than one-third of professional effort to research. Evaluation of clinical investigation graduates who have moved into their research careers can inform program directors about domains of research expertise and methodological areas that may merit additional emphasis in the curriculum.

